# Influence of leptin administration to pregnant female mice on obesity development, taste preferences, and gene expression in the liver and muscles of their male and female offspring

**DOI:** 10.18699/VJ21.076

**Published:** 2021-10

**Authors:** E.I. Denisova, M.M. Savinkova, E.N. Makarova

**Affiliations:** Institute of Cytology and Genetics of the Siberian Branch of the Russian Academy of Sciences, Novosibirsk, Russia; Novosibirsk State University, Novosibirsk, Russia; Institute of Cytology and Genetics of the Siberian Branch of the Russian Academy of Sciences, Novosibirsk, Russia

**Keywords:** leptin, taste preferences, obesity, developmental programming, лептин, вкусовые предпочтения, ожирение, программирование развития

## Abstract

Abstract. The consumption of food rich in sugar and fat provokes obesity. Prenatal conditions have an impact on taste preferences and metabolism in the adult offspring, and this impact may manifest differently in different sexes. An increase in blood leptin level in pregnant females reduces the risk of obesity and insulin resistance in the offspring, although the mechanisms mediating this effect are unknown. Neither is it known whether maternal leptin affects taste preferences. In this study, we investigated the effect of leptin administration to pregnant mice on the development of diet-induced obesity, food choice, and gene expression in the liver and muscles of the offspring with regard to sex. Leptin was administered to female mice on days 11, 12, and 13 of pregnancy. In male and female offspring, growth rate and intake of standard chow after weaning, obesity development, gene expression in the liver and muscles, and food choice when kept on a high-calorie diet (standard chow, lard, sweet cookies) were recorded. Leptin administration to pregnant females reduced body weight in the female offspring fed on the standard diet. When the offspring were given a high-calorie diet, leptin administration inhibited obesity development and reduced the consumption of cookies only in males. It also increased the consumption of standard chow and the mRNA levels of genes for the insulin receptor and glucose transporter type 4 in the muscles of both male and female offspring. The results demonstrate that an increase in blood leptin levels in pregnant females has a sex-specif ic effect on the metabolism of the offspring increasing resistance to obesity only in male offspring. The mechanism underlying this effect includes a shift in food preference in favor of a balanced diet and maintenance of insulin sensitivity in muscle tissues.

## Abstract (ru)

Потребление сладкой и жирной пищи способствует развитию ожирения. Условия пренаталь- ного развития влияют на вкусовые предпочтения и метаболизм в зрелости, и это может по-разному про- являться в зависимости от пола. Показано, что повышение уровня лептина в крови беременных самок сни- жает риск развития ожирения и инсулинорезистентности у потомства, однако механизмы его действия на чувствительность к инсулину у потомства не установлены. Неизвестно также, влияет ли материнский лептин на вкусовые предпочтения. Задачей настоящего исследования было изучение влияния введения лептина беременным самкам мышей на развитие ожирения, индуцированного диетой, вкусовые предпочтения и экс- прессию генов в печени и мышцах у потомства в зависимости от пола. Оценивали влияние введения лептина самкам мышей на 11, 12 и 13-й день беременности на рост и потребление пищи в стандартных условиях, развитие ожирения, выбор компонентов пищи и экспрессию генов в печени и мышцах при содержании на высококалорийной диете (стандартный корм, свиное сало, сладкое печенье) у потомства разного пола. Введение лептина беременным самкам снижало вес тела у женского потомства на стандартной диете. При содержании потомства на высококалорийной диете введение лептина тормозило развитие ожирения и сни- жало потребление печенья только у самцов, а также повышало потребление стандартного корма и уровень мРНК генов инсулинового рецептора и переносчика глюкозы четвертого типа в мышцах у потомства обоего пола. Результаты демонстрируют, что повышение лептина в крови беременных самок оказывает зависящее от пола влияние на метаболизм потомства, увеличивает устойчивость к развитию ожирения только у потом- ства мужского пола и механизм этого влияния включает в себя смещение вкусовых предпочтений в пользу сбалансированного корма и поддержание чувствительности мышц к инсулину.

## Introduction

Nowadays, one of the main causes of the widespread occurrence of obesity and conditions associated therewith is the consumption of high-calorie food (Astrup et al., 2008). The choice of food considerably depends on taste preferences (Duffy et al., 2009), and the preference to palatable fatty and sweet (obesogenic) food contributes to the epidemic prevalence of obesity (May, Dus, 2021; Spinelli et al., 2021).

Taste preferences and tendency to metabolic impairments in an individual are determined by the genotype (Chmurzynska, Mlodzik, 2017; Diószegi et al., 2019) and early development conditions (Mezei et al., 2020). The undernourishment, overnutrition, obesity, and diet of pregnant and nursing dams may exert deferred effects on the taste preferences and metabolic phenotype of the offspring in adulthood and thereby increase or decrease the risk of obesity (Barker, Osmond, 1986; Ong et al., 2012; Gabory et al., 2013; Bale, 2015). It is important to investigate the maternal environment factors that modulate offspring development and the molecular and physiological mechanisms targeted by these factors. Understanding them may add to the elaboration of methods that would correct development in order to reduce the risk of metabolic impairments.

Leptin, a hormone produced in the adipose tissue, is considered to be a programming factor of the maternal environment. Model experiments indicate that high maternal leptin levels in the pregnancy reduce body weight, increase insulin sensitivity, and improve metabolic characteristics in the adult offspring fed on balanced or high-calorie diets, and this effect may manifest differently in different sexes (Pennington et al., 2012; Makarova et al., 2013; Pollok et al., 2015; Talton et al., 2016; Denisova et al., 2020). Nevertheless, molecular mechanisms mediating the programming action of maternal leptin remain obscure.

Maternal leptin may exert a deferred effect on metabolic processes in the liver and muscles of the offspring. Developmental programming is thought to be associated with changes in gene expression in the offspring via epigenetic modifications induced by maternal environmental factors (Laker et al., 2014). It is unknown whether leptin affects the expression of genes encoding regulatory factors and enzymes responsible for carbohydrate and lipid metabolism in the liver and muscles of the offspring. Maternal leptin may also contribute to a lower predisposition to diet-induced obesity in the offspring via food choice (Pollock et al., 2015), but this issue has been poorly studied. The diet components (fats, proteins, or sugars) the consumption of which may be affected by maternal leptin are unknown. Neither is it known whether the programming action of maternal leptin on the offspring depends on sex or what mechanisms mediate the programming effect of maternal leptin on food choice in the offspring.

The goal of this study was to investigate the effect of leptin administration to pregnant female mice on metabolic indices, taste preferences, and the expression of genes in the liver and muscles of the offspring with regard to sex.

## Materials and methods

**Experimental animals.** The European Parliament Directive 2004/10/EC on the principles of good laboratory practice and the Russian regulations for accommodation and care of animals, GOST 33215-2014, were followed.

Experiments were conducted with С57BL/6J mice housed at the vivarium of the Institute of Cytology and Genetics, Novosibirsk, Russia. The animals were kept at 12-h daylight with free access to water and standard chow for the conventional maintenance and breeding of rodents (BioPro Company, Novosibirsk, Russia). Mature females were mated to males of the same strain. The mating was judged from the presence of a copulation plug. The appearance of the plug signified day 0 of pregnancy. The females were administered 0.2 mg/kg of recombinant murine leptin (Peprotech, United Kingdom) or the same volume of normal saline on days 11, 12, and 13 of pregnancy. The injections were done subcutaneously in the shoulder area. The female weight and amount of consumed food were recorded daily. The delivery dates and litter sizes were recorded. Large litters (> 7) were reduced to 7 on day 1 after delivery by discarding pups of the lowest weights. The dam and pups were weighted on days 0, 7, 14, 21, and 28 after delivery. The offspring were separated from their mothers on day 28 after birth.

To assess the effect of maternal leptin on the metabolic indices of the offspring in the postnatal life, one male and one female from each litter were kept individually. The female offspring of dams having received normal saline included 6 animals, and the male, 5. The female offspring of dams having received leptin was 8 animals, and so was the male offspring. The animals were weighted on a weekly basis, and the amount of food eaten weekly was assessed. At the age of 10 weeks, the mouse diet was supplemented with obesogenic food: sweet cookies and pork lard. Mice were being fed on this diet for 10 weeks and were weighted weekly. The standard chow was replaced once a week, and cookies and lard were replaced three times a week. The following parameters were recorded: the amounts of daily eaten standard chow, cookies, and lard; the amounts of energy consumed with these kinds of food (lard, 8 kcal/g; standard chow, 2.5 kcal/g; cookies, 4.58 kcal/g); the overall amount of consumed energy normalized to body weight; and the energy consumed with each kind of food as a percentage of the entire energy consumed.

At the end of the experiment, mice were euthanized by decapitation. Muscle and liver tissue samples were frozen and stored in liquid nitrogen to measure the rate of gene expression.

**Diet.** Standard chow was purchased from BioPro, Novosibirsk, Russia. Composition: two-component grain mixture, milk components, high-protein components (vegetable and animal proteins), vegetable oil, amino acids, organic acids, vitamin-mineral premix, and fiber. Crude protein: 22 %. Energy value 2500 kcal.

Pork lard and cookies were bought in a food store. Cookie composition (g/100 g): proteins – 6.9, fats – 18.4, carbohydrates – 71.8. Energy value 458 kcal/100 g. Lard (subcutaneous fat): proteins – 1.8, fats – 94.2, carbohydrates – 0. Energy value 800 kcal/100 g.

**Assay of mRNAs.** Levels of mRNAs were assessed by reverse transcription followed by relative quantitation real-time PCR. Total RNA was isolated from tissues with an ExtractRNA kit (Evrogen, Moscow, Russia) according to manufacturer’s recommendatPCR was conducted with the qPCRmix-HS LowROX reaction premix (Evrogen) and the TaqMan Gene Expression Assay system for mouse genes (Applied Biosystems): Insr, Mm01211875_m1; Fgf 21, Mm00840165_g1; G6pc, Mm00839363_m1; GCk, Mm00439129_m1; Ppargc1a, Mm01208835_m1; Pklr, Mm00443090_m1; Acaca, Mm01304257_m1; Pnpla2, Mm00503040_m1; Ig f, Mm00439560_m1; Slc2a4, Mm00436615_m1; and Actb, Mm00607939_s1.ions. Reverse transcription was conducted with MMLV reverse transcriptase (Evrogen) and oligo-dT primer according to manufacturer’s recommendations.

The expression rates of the following genes were assayed in the liver: InsR for insulin receptor, Gck for glucokinase, Pklr for pyruvate kinase, G6pc for glucose-6-phosphatase, Pnpla2 for triacylglyceride lipase ATGL, Acaca for acetyl Co-A carboxylase, Pgc1 for peroxisome proliferator-activated receptor γ coactivator PPARGC1A, Fg f 21 for fibroblast growth factor 21, and Ig f1 for insulin-like growth factor 1. In muscles: Slc2a4 for glucose transporter GLUT4 and InsR. The Actb gene for β-actin was used for reference.

The results were statistically evaluated with Statistica 10.0. Descriptive statistics was used in the calculation of group means and standard errors of the mean. Body weight and food consumption in pregnant females and their offspring were analyzed by repeated measures ANOVA with the assessment of the factors “experimental treatment” (administration of leptin or normal saline) and “day of pregnancy” for pregnant females; factors “sex” (males or females), “experiment” (administration of leptin of normal saline to mothers), and “age” (4 to 10 weeks) for the offspring fed on standard diet; factors “sex”, “experiment”, and “age” (10 to 20 weeks) for the offspring fed on the obesogenic diet. Intergroup differences were assessed by the Newman–Keuls post-hoc test. The effects of sex and the prenatal factor on gene expression in the liver and muscles were assessed by two-way ANOVA with the “sex” and “experiment” factors. Intergroup differences were assessed with Student’s t test. The results are shown in plots as mean ± SEM.

## Results

**Fig. 1.**
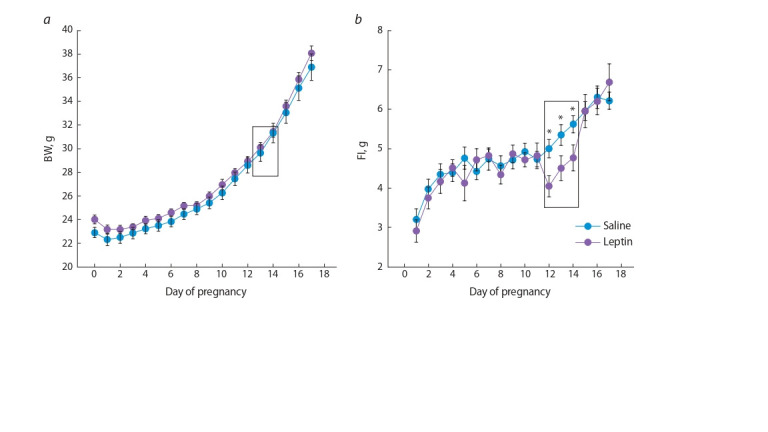
Influence of leptin administered to female mice on pregnancy days 11, 12, and 13 on body weight (a) and food intake (b). Data are presented as mean ± SEM. *p < 0.05, Student’s t test.

**Influence of leptin administration on food consumption and body weight in pregnant females.** Leptin administration to female mice on pregnancy days 11, 12, and 13 had no significant impact on body weight (Fig. 1, a), although it reduced food intake by 20 % ( p < 0.001, repeated measures ANOVA, factors “experiment” × “day of pregnancy” (see Fig. 1, b). The anorectic action of leptin lasted for no more than 24 h, as the amounts of food consumption by females having received leptin and normal saline aligned on day 2 after the last injection.

**Influence of leptin administered to female mice on body weight and the consumption of standard chow in the male and female offspring.** When nursed, the male and female offspring did not differ in weight. Leptin administration to pregnant dams had no significant effect on offspring weight (data not shown).

The dynamic patterns of weight after weaning onto standard chow were different in males and females. Within the first week of feeding on standard chow, males overweighed females and weighed more throughout the experiment ( p < 0.0001, F1.24 = 30.32, “sex”, “sex” × “age” p < 0.00001, F6.144 = 8.23, repeated measures ANOVA). We performed repeated measures ANOVA with the “experiment” and “age” (weeks 4–10) factors in males and females separately.

**Fig. 2.**
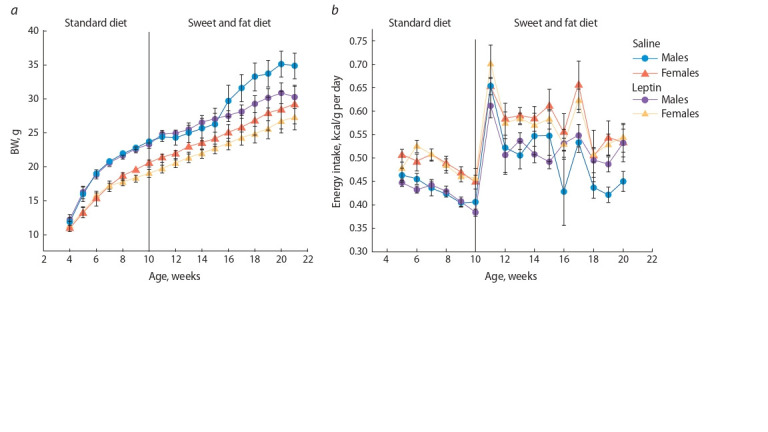
Influence of leptin administration to pregnant female mice on body weight (a) and the ratio of consumed energy and body weight (b) in the male and female offspring receiving the standard and obesogenic diet.

The male offspring whose mothers had received leptin did not differ in weight from the male offspring of control females when kept singly and fed on standard chow in weeks 4–10. The female offspring of leptin-receiving females did not differ from the female offspring of control females till sexual maturity (8 weeks), but then they lagged behind females born in the control group, and this trend remained throughout the experiment (Fig. 2, a). Females consumed more energy per body weight unit than males ( p < 0.0001, F1.24 = 34.1, factor “sex”, repeated measures ANOVA). Leptin administration to mothers during pregnancy did not influence this index (see Fig. 2, b).

**Body weight and food consumption in the male and female offspring fed on obesogenic diet.** Repeated measures ANOVA revealed no effect of the “experiment” factor on the female offspring in the analysis of body weights of males and females fed on obesogenic diet within 10 weeks. However, it showed a significant interaction between the “age” and “experiment” factors ( p < 0.01, F9.90 = 2.82) in the male offspring. The male offspring of dams having received normal saline or leptin did not differ in body weight, but then the males born in the control group began to gain weight sharply and outperformed the males born to leptin-receiving dams (see Fig. 2, a). In the females born to control and leptin-receiving dams, the difference arising in keeping on standard chow remained when they received the obesogenic diet (see Fig. 2, a).

Energy consumption normalized to body weight increased dramatically when lard and cookies were added to the diet (see Fig. 2, b). It remained higher in females than in males ( p < 0.01, F1.22 = 9.06, factor “sex”, repeated measures ANOVA).

**Fig. 3.**
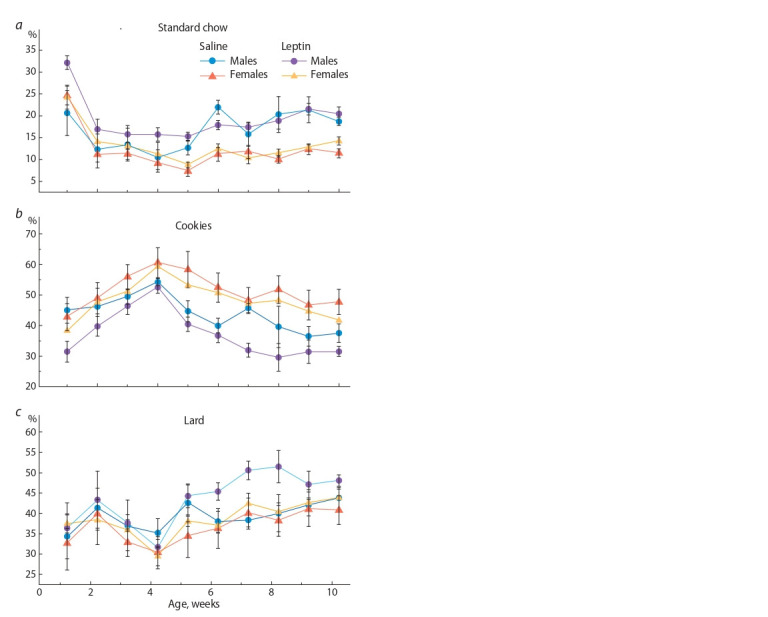
Influence of leptin administered to pregnant mice on the percentages of energy consumed with standard chow, cookies, and lard in the male and female offspring fed on the obesogenic diet.

To assess the influence of maternal leptin on taste preferences in the offspring, the contributions of each diet component (standard chow, lard, and cookies) to the overall energy consumption were analyzed. Pronounced sex differences in the consumption of standard chow (males ate more than females, p < 0.001, F1.22 = 34, factor “sex”) and cookies (males ate less than females, p < 0.01, F1.22 = 12.2, factor “sex”) were recorded. No significant difference between males and females in lard consumption was noted (Fig. 3).

Leptin administration to pregnant mice affected taste preferences in the offspring, and this effect was more pronounced in males. Maternal leptin increased the share of standard chow in the overall energy consumption in the offspring of both sexes within the first five weeks of receiving the obesogenic diet ( p < 0.05, F1.22 = 4.03, factor “experiment”, repeated measures ANOVA), but in males the effect was greater than in females (see Fig. 3, a). Maternal leptin lowered the share of cookies only in males throughout the time of being fed on obesogenic diet ( p < 0.05, F1.22 = 5.3, factor “experiment”, repeated measures ANOVA, Fig. 3, b) and had no impact on lard consumption regardless of sex (see Fig. 3, c).

**Fig. 4.**
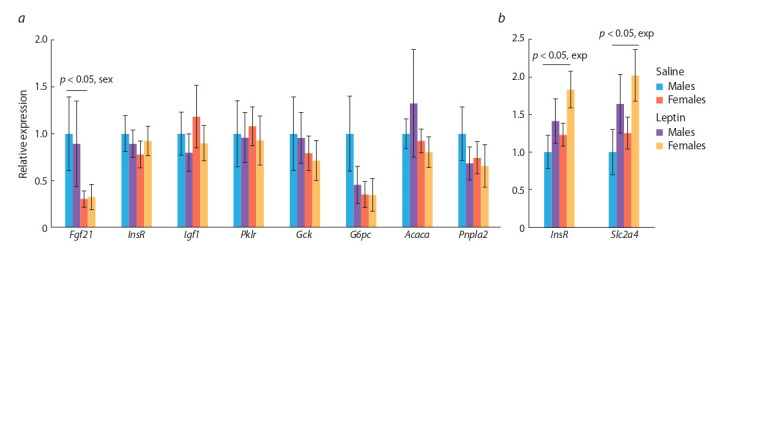
Influence of leptin administered to pregnant mice on gene expression in the liver (a) and muscles (b) of the male and female offspring fed on the obesogenic diet.

**Expression of genes involved in carbohydrate and lipid metabolism in the liver and muscles.** The expression rates of the following genes were assayed in liver tissue: InsR for insulin sensitivity, Gck and Pklr for glycolysis enzymes, G6pc for gluconeogenesis, Pnpla2 for lipolysis (adipose triglyceride lipase), Acaca for lipogenesis, and genes encoding regulatory factors that affect metabolism in the liver (Pgc1) or are involved in the regulation of carbohydrate and lipid metabolism in the entire body (Fg f 21 and Ig f1). Leptin administration to pregnant mice did not affect the expression of these genes in the liver. A significant influence of sex on the expression of the Fg f 21 gene for fibroblast growth factor 21 was noted. Its mRNA level was lower in females than in males ( p < 0.05, F1.18 = 5.4, 2-way ANOVA, Fig. 4, а).

In muscles of both sexes, leptin administration to mothers caused higher expression rates of the genes InsR for insulin receptor and Slc2a4 for insulin-dependent glucose transporter ( p < 0.05, 2-way ANOVA in both cases, see Fig. 4, b), pointing to elevated insulin sensitivity in muscles.

## Metabolic indices

Females had lower liver weights than males ( p = 0.051, F1.18 = 4.35, 2-way ANOVA) and lower blood cholesterol levels ( p < 0.001, F1.18 = 49.82, 2-way ANOVA). They did not differ from males in blood glucose or triglyceride levels (see the Table). Leptin administration to pregnant mothers did not affect the metabolic indices tested in either males or females.

**Table 1.**
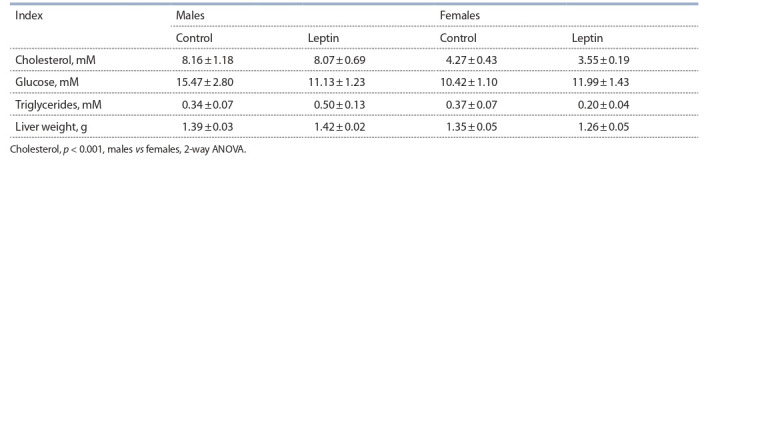
Inf luence of leptin administration to pregnant mice on liver weight and blood biochemistry in male and female offspring fed on the obesogenic diet

## Discussion

Previous works with mice and rats demonstrated that an increase in maternal blood leptin level increased the resistance to diet-induced obesity in the offspring, and the programming action of maternal leptin might depend on the offspring sex (Stocker et al., 2007; Makarova et al., 2013). Here, we tested the hypothesis that the said effect of maternal leptin was associated with its influence on taste preferences and on gene expression in the liver and muscles. For this purpose, we administered leptin to females at the minimum threshold dose inducing physiological response (Enriori et al., 2007) on days 11, 12, and 13 of pregnancy and assessed taste preferences, metabolic indices, and gene expression in the liver and muscles in the offspring of both sexes. We chose this time interval of pregnancy because sex differentiation in mouse fetuses starts on days 11, 12 (Hacker et al., 1995). Just on day 12, the proliferation of precursors of hypothalamic neurons that will control energy consumption and expenditure reaches its maximum (Ishii, Bouret, 2012).

Earlier we found that a single leptin administration to mice on pregnancy day 12 exerts sex-specific programming action on metabolism in the offspring (Denisova et al., 2020). Thus, females are sensitive to leptin in this period, as evident from lower food consumption in response to leptin. At later pregnancy stages, leptin sensitivity in females may decrease as a result of the significant increase in endogenous blood leptin in the third trimester of pregnancy (Makarova et al., 2010).

Leptin administration to females slowed down the development of diet-induced obesity in the male offspring, which agrees with earlier studies, where leptin administration to female rats from day 14 of pregnancy till the end of lactation (Stocker et al., 2007) and to mice at the end of pregnancy (Makarova et al., 2013) prevented diet-induced obesity in the male offspring fed on obesogenic diet. A single injection of leptin on day 12 of pregnancy prevented hyperglycemia in the offspring with obesity and tended to decrease the rate of diet-induced obesity development in the male offspring (Denisova et al., 2020). However, in contrast to the data of this work, a single leptin administration to female mice on day 12 of pregnancy did not affect the growth rate of the female offspring fed on standard chow. Probably, the leptin administration on day 12 only left intact the initial steps of sex differentiation, which might be leptin-sensitive.

This study shows that maternal leptin may affect taste preferences in the offspring, and this effect may be one of the factors causing resistance to diet-induced obesity in the male pups with free choice of components of obesogenic diet. This result is new. Females consumed more cookies and less standard chow than males. This observation is consistent with the sex differences in consuming sweet food observed in various species (Valenstein et al., 1967; Zucker et al., 1972; Buczek et al., 2020). The causes of these differences are sought in the influence of biological sex on central systems regulating energy homeostasis and on reward systems (Sinclair et al., 2017; Buczek et al., 2020).

Our results are the first to demonstrate explicitly that the liver hormone FGF21 may be involved in the sex-dependent regulation of taste preferences. The expression of Fg f 21 in the livers of females was significantly lower than in males, which is in agreement with earlier data (Bazhan et al., 2019). The blood FGF21 level correlates with the expression rate of its gene in the liver. It is elevated in obese males (Bazhan et al., 2019). It has been shown that FGF21 increases protein consumption (Larson et al., 2019) and decreases sugar consumption (Talukdar et al., 2016); thus, the higher FGF21 level in males as compared to females may be the cause of higher consumption of standard protein-rich chow and lower consumption of sweet cookies in males than in females.

Leptin administration to pregnant females decreased cookie consumption and increased standard chow consumption in the offspring. Similar results were obtained in experiments by K.E. Pollock et al. (2015) with mice. They found that hyperleptinemia in pregnant females shifted taste preferences in the offspring towards higher consumption of standard chow as compared to sweet food.

The mechanisms underlying this effect are unknown, and our work indicates that they are not associated with Fg f 21 expression in the liver, because leptin administration to pregnant mice did not change the Fg f 21 mRNA level in the offspring. It is conceivable that the programming action of maternal leptin is associated with its influence on the motivation and reward systems and systems regulating food behavior in the offspring.

It has been shown that maternal environment factors during pregnancy and nursing may affect the motivation and reward systems in the offspring, which involve endogenous opioids, dopamine, and serotonin (Grissom et al., 2014). The type of leptin influence on the development of food consumption regulation systems demands further studies, and our results indicate that the embryo development period from day 11 to 13 is the window in which these systems are susceptible to maternal environment factors.

O.O. Talton et al. (2016) showed that high blood leptin in pregnant mice increased insulin sensitivity in the offspring regardless of diet. We found no effect of maternal leptin on the expression of liver genes involved in glucose metabolism (InsR, Ig f1), fatty acid oxidation (Fg f 21), glycolysis (Gck), or gluconeogenesis (Pklr, G6pc). Neither did it affect the expression of genes for lipolysis (Atgl ) or lipogenesis (Acaca). Our results demonstrate that the effect of maternal leptin increasing insulin sensitivity may be mediated by the expression of genes regulating glucose metabolism in muscles of the offspring. The expression of genes for insulin receptor (Insr) and insulindependent glucose transporter (Slc2a4) in muscles was higher in the offspring of leptin-receiving females as compared to control ones. Apparently, maternal leptin supports insulin sensitivity in the consumption of obesogenic food by the offspring, which may also work against obesity development.

The molecular mechanisms underlying the programming action of maternal leptin are obscure. It is unknown whether maternal leptin penetrates to the fetus bloodstream through placenta. However, at high leptin levels in pregnant females it may penetrate through placenta, as we found in our previous studies that the blood plasma leptin levels increased manifold in both the females and the fetuses within one hour after its administration at the end of pregnancy (Denisova, Makarova, 2018). Also, it has been shown that leptin administration to pregnant mice reduces the weights of fetuses and placentae (Yamashita et al., 2001; Denisova et al., 2020). The placentae of male and female fetuses respond to leptin administration differently: in male fetuses, leptin reduces placenta weight and in female ones, the expression of glucose transporters in placentae (Denisova et al., 2020). The sex-specific programming action of maternal leptin on metabolism in the offspring may be mediated by its different effects on placenta functions in fetuses of different sexes. More studies are demanded for understanding the mechanisms by which maternal leptin affects fetus development.

## Conclusion

We show that triple leptin administration to females on pregnancy days 11, 12, and 13 delayed diet-induced obesity development in the male offspring. It also shifted taste preferences towards the consumption of balanced diet and increased the expression rates of genes for insulin receptor and insulindependent glucose transporter in muscles in the offspring of both sexes. These results suggest that maternal leptin increases the resistance to diet-induced obesity in the offspring via taste preferences and higher muscle sensitivity to insulin.

## Conflict of interest

The authors declare no conflict of interest.
